# Secrecy Energy Efficiency Enhancement in UAV-Assisted MEC System

**DOI:** 10.3390/s23020723

**Published:** 2023-01-08

**Authors:** Jiansong Miao, Haoqiang Chen, Hairui Li, Shanling Bai

**Affiliations:** School of Information and Communication Engineering, Beijing University of Posts and Telecommunications, Beijing 100876, China

**Keywords:** unmanned aerial vehicle communication, mobile edge computing, secrecy energy efficiency, convex optimization algorithm

## Abstract

A secrecy energy efficiency optimization scheme for a multifunctional unmanned aerial vehicle (UAV) assisted mobile edge computing system is proposed to solve the computing power and security issues in the Internet-of-Things scenario. The UAV can switch roles between a computing UAV and jamming UAV based on the channel conditions. To ensure the security of the content and the system energy efficiency in the process of offloading computing tasks, the UAV trajectory, uplink transmit power, user scheduling, and offload task are jointly optimized, and an updated-rate assisted block coordinate descent (BCD) algorithm is used. Simulation results show that this scheme efficiently improves the secrecy performance and energy efficiency of the system. Compared with the benchmark scheme, the secrecy energy efficiency of the scheme is improved by 38.5%.

## 1. Introduction

With the growing number of intelligent mobile devices, computing intensive applications such as video calling and virtual reality are becoming more common. However, while providing convenience, mobile devices also have the disadvantage of limited computing resources, which makes it a challenge to provide a high level of experience quality. Mobile edge computing (MEC) has also received widespread attention [1]. Mobile edge computing significantly reduces network overhead and task execution delay [2]. Traditional network edge servers are located in base stations all over the communication network. However, fixed base stations still have drawbacks such as limited coverage and severe link loss.

Unmanned aerial vehicles (UAVs) have the characteristics of small size, low cost, rapid deployment on demand, and high-probability air-to-ground line-of-sight (LOS) link, which produces high channel gain in the communication system. Therefore, a large number of studies considered introducing UAVs equipped with computing equipment into mobile edge computing networks to assist ground users in completing computing tasks. Hu et al. [3] established a UAV-assisted mobile edge computing system. The UAV provides computing services for multiple users in the form of orthogonal multiple access. Zhang et al. [4] studied the computational efficiency of a UAV MEC system and maximized the computational efficiency by jointly optimizing the offloading time, CPU frequency, user transmit power, and UAV trajectory. Yu et al. [5] minimized the service delay of all IoT devices and the weighted sum of UAV energy consumption by jointly optimizing UAV location, communication, computing resource allocation, and task splitting decisions. In the research of Shang et al. [6], the ground computing server and the computing UAV provide computing resources for the ground terminal at the same time. To minimize the total energy consumption of the ground terminal, user scheduling, uplink power control, channel allocation, computing task allocation, and the 3D layout of UAVs are jointly optimized. Kumar et al. [7] proposed a drone assisted distributed routing framework focusing on quality of service provision in IoT environments (D-IoT). The aerial drone mobility and parameters are modeled probabilistically, focusing on highly dynamic flying ad hoc networks environments. A comparative performance evaluation attests to the benefits of the proposed drone-assisted routing framework. Kumar et al. [8] developed a complete communication framework for quality of service provisioning in UAV-assisted aerial ad hoc networking (QSPU) based on the aerial ad hoc mobility model and service parameters. Jha et al. [9] presents a noble, innovative idea for designing and developing a blended-wing-body (BWB)-based configuration system for UAVs, especially for next-generation high-speed drone use cases.

Although UAVs assist ground users in completing computing tasks, which greatly reduces computing delays, the process of users offloading computing data from UAVs also has the risk of data leakage, which brings new challenges to the security performance of mobile edge computing systems. Zhou et al. [10] studied the physical layer security of UAV base stations transmitting confidential information to multiple information receivers with the assistance of UAV jammers. By formulating UAV trajectory planning and power control schemes, the minimum average secrecy rate of all receivers was maximized. Zhou et al. [11] used full-duplex legitimate UAVs and non-offloading ground users to transmit jamming signals in order to resist the stealing of the offloading task content by multiple aerial eavesdropping UAVs. In addition, they designed a low-complexity iterative algorithm to maximize the security capacity of the system. Xu et al. [12] studied a dual UAV-assisted mobile edge computing system in which one UAV is used to help the ground terminal equipment computing offloading tasks, and the other is used as a jammer to suppress malicious eavesdroppers. However, two UAVs can complete only one computing and jamming task, and there are limitations to the improvement of system performance.

In addition, the battery capacity of UAVs is limited, which greatly limits the continuous operation time of the system. Zeng et al. [13] established a propulsion power consumption model of a rotary-wing UAV and jointly optimized the trajectory of the UAV and communication time allocation between each ground node to meet the communication throughput requirements of each ground node. In addition, the total energy consumption of the system was minimized. Zhang et al. [14] studied the energy efficiency of a NOMA-based UAV MEC system, mathematically evaluated the energy cost of the system by establishing a utility function, and designed a joint optimization scheme to minimize the energy cost. Zhang et al. [15] studied the task offloading problem in UAV-assisted MEC. To balance user utility and UAV energy consumption, a matching game algorithm with a bilateral preference list was proposed to save energy.

Although research on UAV-assisted mobile edge computing systems has been relatively comprehensive, few studies have considered the system security rate and system energy efficiency at the same time. Although Hua et al. [16] established a system secrecy energy efficiency model and maximized it by jointly optimizing the UAV’s trajectory, transmission power, and user scheduling, they only studied the UAV base station communication system.

Therefore, this paper establishes a MEC system model based on a multi-functional UAV in which the UAV can switch between the auxiliary computing UAV and active jamming UAV according to the channel conditions. By considering both security and energy consumption issues at the same time, with the goal of maximizing the security energy efficiency of the system, the user scheduling, UAV trajectory, uplink power, and offload task volume are jointly optimized to improve the security energy efficiency performance of the system. Compared with previous studies, the models used in this study are quite different with different constraints, and the algorithm solution ideas are similar, but the differences in models and constraints also make the algorithms different.

The main contributions of this work are summarized as follows:

- We propose a MEC system model based on multi-functional UAVs. Two UAVs with both computing and jamming functions cooperate as ground sensor nodes to provide a safe unloading environment and computing services, and UAVs can switch roles between auxiliary computing UAVs and active jamming UAVs according to their geographical locations in different time slots. Under the premise of considering the system energy efficiency, the mathematical model of the communication system security energy efficiency is established. The system security energy efficiency is maximized through the joint optimization of user scheduling, UAV trajectory, uplink transmission power, and computing task allocation in the system.
- In order to deal with the complex non-convex problem, we divide the original problem into two sub-problems. However, the sub-problems with non-convex and fractional objectives are still difficult to deal with. Therefore, the sub-problem is converted into a convex form by applying SCA technology, and the Dinkelbach algorithm solves the fractional problem in an iterative manner. For the solution of two sub-problems, we introduce the BCD method to solve the sub-problem iteratively in an alternate manner until the algorithm converges.
- The system SEE performance and security rate performance of the proposed scheme are verified through simulation, and the UAV trajectory and user scheduling after joint optimization are displayed and analyzed. The comparison of different optimization schemes in the simulation results shows the effectiveness of the proposed joint optimization scheme.

## 2. System Model

**Figure 1:**
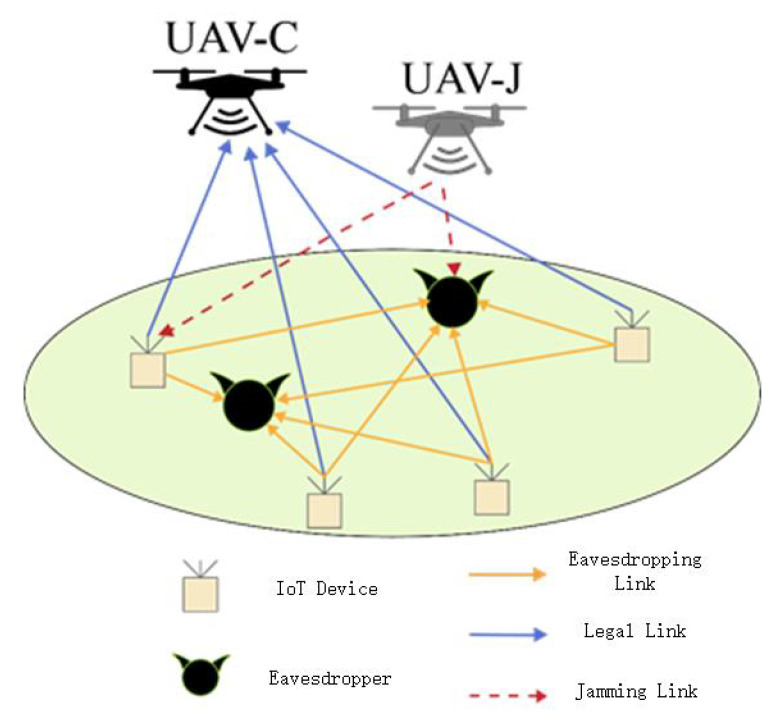
Model of UAV-assisted mobile edge computing system.

Figure 1 depicts a model of a multi-UAV-assisted mobile edge computing system. There are two UAVs in the system, both equipped with transceiver communications and computing devices, which are both capable of assisting in computing and sending signals. According to the channel conditions of its own location, the UAV chooses to act as an auxiliary computing UAV or a friendly jamming UAV, but both kinds of UAVs must exist in the same time slot. Among them, the auxiliary computing UAV is responsible for receiving part of the computing tasks offloaded by *L* ground nodes to help it complete the calculation work, while the friendly jamming UAV is responsible for sending jamming signals to resist the threat of K≥1 ground eavesdropping nodes. In addition, to obtain a more essential relationship between system indicators and system performance, it is assumed that the positions of ground sensor nodes and eavesdropping nodes are fixed, and the two UAVs know the location information of ground nodes and other UAVs. Table 1 describes the meaning of the symbols used in this document.

### 2.1. Channel Model

In this model, *L* ground legal sensor nodes are distributed in an area, and a three-dimensional Cartesian coordinate system is established with the lower left boundary point of the area as the origin. Let the coordinate of the *l*th ground sensor node $I_{l}$ be $w_{I_{l}}={\left[x_{I_{l}},y_{I_{l}}\right]}^{T}$, and let the coordinate of the *k*th ground eavesdropper $E_{k}$ be $w_{E_{k}}={\left[x_{E_{k}},y_{E_{k}}\right]}^{T}$. To record the position change of the UAV within the cycle time, the entire cycle time *T* is discretized into *N* equal time slots, satisfying T=Nδ, where δ is the length of the unit time slot. Since δ is small enough, it is assumed that the UAV is at a fixed position in each time slot [17]. In addition, it is assumed that the flight height of the UAV is fixed as *H*, where it can avoid collisions with buildings on the ground. By setting the position coordinates of the UAV in each time slot, the trajectory of the UAV can be approximated as a line segment connected by *N* discrete points, and the position of UAV $U_{m}$ in the *n*th time slot can be expressed as $q_{U_{m}}\left[n\right]={\left[x_{U_{m}}\left[n\right],y_{U_{m}}\left[n\right]\right]}^{T}$ with $m=\left[1,2\right],n\in \left[1,N\right]$. Then, the trajectory of the UAV needs to satisfy the following constraints:

(1) $$\begin{array}{c} \|q_{U_{m}}\left[n+1\right]-q_{U_{m}}\left[n\right]{\|}^{2}\leq V_{\max}^{2}\delta^{2};n=1,\ldots ,N-1 \end{array}$$

(2) $$\begin{array}{c} q_{U_{m}}\left[1\right]=q_{U_{m}0}; \end{array}$$

(3) $$\begin{array}{c} q_{U_{m}}\left[N\right]=q_{U_{m}F}; \end{array}$$

where the constraints in (1) indicate that the maximum distance of the UAV within time length δ is limited by the maximum flight rate $V_{\max}$ of the UAV, (2) and (3) restrict the start and end position coordinates of each UAV, $q_{U_{m}0}$ represents the initial position of $U_{m}$, and $q_{U_{m}F}$ represents the termination position of $U_{m}$. In addition, due to the small size of the UAVs, collisions between UAVs are ignored.

The study selects a relatively simple path-loss model close to the actual situation, so it is assumed that the Doppler effect caused by high-speed movement can be perfectly compensated, and the two channels other than the ground–ground link are regarded as line-of-sight links, which satisfies the free-space loss model. Therefore, the channel power gain of the link between the ground sensor node and auxiliary computing UAV in the time slot can be expressed as

(4a) $$\begin{array}{c} h_{I_{l}U_{m}}\left[n\right]=\rho_{0}d_{I_{l}U_{m}}^{-2}\left[n\right]=\frac{\rho_{0}}{∥q_{U_{m}}\left[n\right]-w_{I_{l}}\left[n\right]{∥}^{2}+H^{2}} \end{array}$$

where $\rho_{0}$ is the channel power when the distance is referenced $d_{0}=1m$, and $d_{I_{l}U_{m}}\left[n\right]$ indicates the distance between the two nodes in the *n*th time slot. Similarly, the channel power gain of the link between jamming UAV $U_{m}\;$ and ground eavesdropping node $E_{k}$ in the *n*th time slot is

(4b) $$\begin{array}{c} h_{U_{m}E_{k}}\left[n\right]=\rho_{0}d_{U_{m}E_{k}}^{-2}\left[n\right]=\frac{\rho_{0}}{∥q_{U_{m}}\left[n\right]-w_{E_{k}}\left[n\right]{∥}^{2}+H^{2}} \end{array}$$

where $d_{U_{m}E_{k}}\left[n\right]$ represents the distance between the jamming UAV and the eavesdropping node in the *n*th time slot. The channel power gain of the air–air link of the two UAVs in the *n*th time slot can be expressed as

(4c) $$\begin{array}{c} h_{U_{1}U_{2}}\left[n\right]=\rho_{0}d_{U_{1}U_{2}}^{-2}\left[n\right]=\frac{\rho_{0}}{∥q_{U_{1}}\left[n\right]-q_{U_{2}}\left[n\right]{∥}^{2}} \end{array}$$

where $d_{U_{1}U_{2}}\left[n\right]$ represents the distance between the two UAVs in the *n*th time slot. Ground sensor node $I_{l}$ and the eavesdropping node $E_{k}$ obey Rayleigh fading, and the channel gain is

(4d) $$\begin{array}{c} h_{I_{l}E_{k}}=\rho_{0}d_{I_{l}E_{k}}^{-3}\xi =\frac{\rho_{0}\xi }{∥w_{I_{l}}-w_{E_{k}}{∥}^{3}}\; \end{array}$$

where ξ denotes the Rayleigh fading coefficient, which obeys the exponential distribution of the unit mean, and $d_{I_{l}E_{k}}$ denotes the distance between two ground nodes.

### 2.2. Secrecy Rate Model

In this study, a time-division protocol based on time-division multiplexing is used, where each time slot δ is divided into *L* subtime slots and assigned to each ground sensor node in sequence. Defining the time allocation variable as $\tau_{I_{l}}\left[n\right]$, the offloading duration of the ground sensor node $I_{l}$ in the *n*th time slot can be expressed as $\tau_{I_{l}}\left[n\right]\delta$. To ensure the rationality of the time allocation variables, $\tau_{I_{l}}\left[n\right]$ shall meet the following constraints:

(5a) $$\begin{array}{c} \underset{l=1}{\sum^{L}}\;\tau_{I_{l}}\left[n\right]\leq 1\; \end{array}$$

(5b) $$\begin{array}{c} 0\leq \tau_{I_{l}}\left[n\right]\leq 1;l=1,\ldots ,L \end{array}$$

In addition, the power of the UAV to send the jamming signal is defined as $P_{J}$, and $P_{J}>0$. Define the offloaded transmission power of the ground sensor node $I_{l}$ in the *n*th time slot as $P_{I_{l}}\left[n\right]$ and satisfy the following constraints:

(6) $$\begin{array}{c} 0\leq P_{I_{l}}\left[n\right]\leq P_{\max} \end{array}$$

where $P_{\max}$ denotes the maximum transmit power of the UAV. The transmission rate between the ground node $I_{l}$ and the auxiliary computing UAV in the *n*th time slot can be expressed as

(7) $$\begin{array}{c} R_{I_{l}}\left[n\right]=\tau_{I_{l}}\left[n\right]\log_{2}\left(1+\frac{P_{I_{l}}\left[n\right]\sum_{m=1}^{2}a_{m}\left[n\right]h_{I_{l}U_{m}}\left[n\right]}{\sigma^{2}+P_{J}h_{U_{1}U_{2}}\left[n\right]}\right) \end{array}$$

where $\sigma^{2}$ denotes the Gaussian white noise at the receiver, and $a_{m}\left[n\right]$ denotes whether $U_{m}$ in the *n*th time slot is working in the auxiliary computing mode; $a_{1}\left[n\right]=1,a_{2}\left[n\right]=0$ denotes that $U_{1}$ in the *n*th time slot is the auxiliary computing UAV, and $U_{2}$ is the active jamming UAV. $a_{1}\left[n\right]=0,a_{2}\left[n\right]=1$ denotes that $U_{1}$ in the $n_{th}$ time slot is the active jamming UAV, and $U_{2}$ is the auxiliary computing UAV.

Similarly, the transmission rate between eavesdropper $E_{k}$ and ground node $I_{l}$ in the *n*th time slot is expressed as

(8) $$\begin{array}{c} R_{E_{k}I_{l}}\left[n\right]=\tau_{I_{l}}\left[n\right]\log_{2}\left(1+\frac{P_{I_{l}}\left[n\right]h_{E_{k}I_{l}}}{\sigma^{2}+\sum_{m=1}^{2}\left(1-a_{m}\left[n\right]\right)P_{J}h_{U_{m}E_{k}}\left[n\right]}\right) \end{array}$$

In this study, the secure transmission rate of each link is defined by the difference between the transmission rate of legal links and illegal links. Then, in the *n*th time slot, the uplink transmission rate of sensor node $I_{l}$ is expressed as

(9) $$\begin{array}{c} R_{l}^{\sec}\left[n\right]={\left[R_{I_{l}}\left[n\right]-\max_{\forall k}\;R_{E_{k}I_{l}}\left[n\right]\right]}^{+} \end{array}$$

where ${\left[x\right]}^{+}$ denotes the larger value in 0, and *x*, ${\left[x\right]}^{+}=\max\left(x,0\right)$.

### 2.3. Edge Computing Model

Let $c_{l}$ denote the number of CPU cycles required to calculate 1 bit of data at the *l*th sensor node, and let $D_{l}^{\mathrm{loc}}\left[n\right]$ denote the number of bits that the *l*th sensor node completes the calculation locally in the *n*th time slot. The following constraints are met:

(10) $$\begin{array}{c} c_{I}D_{l}^{\mathrm{loc}}\left[n\right]\leq \delta F_{I}^{\max} \end{array}$$

where $F_{I}^{\max}$ denotes the maximum CPU frequency of sensor node $I_{l}$.

Assume that the computing UAV executes the computing task immediately after receiving each bit of data and must complete the task within its assigned data upload time $\delta \tau_{I_{l}}\left[n\right]$. Since the amount of data in the calculation results is far less than the amount of data actually unloaded [18], the time for retrieving the calculation results is ignored here. The computing power of the computing UAV should satisfy the following constraints:

(11) $$\begin{array}{c} c_{U}B\delta R_{l}^{\sec}\left[n\right]\leq \delta \tau_{I_{l}}\left[n\right]F_{U}^{\max} \end{array}$$

where *B* denotes the channel bandwidth, $c_{U}$ indicates the number of CPU cycles required for the UAV to calculate 1 bit of data in the auxiliary computing, and $F_{U}^{\max}$ indicates the maximum CPU frequency of the UAV to ensure that the total number of CPU cycles required for UAV calculation in each time slot does not exceed its maximum computing power.

$Q_{l}$ is defined to represent the minimum amount of data required by the *l*th sensor node in each time slot, so the following constraints should be satisfied:

(12) $$\begin{array}{c} D_{l}^{\mathrm{loc}}\left[n\right]+B\delta R_{l}^{\sec}\left[n\right]\geq Q_{l} \end{array}$$

### 2.4. Energy Consumption Model

UAVs can be divided into fixed wing UAVs and rotary wing UAVs according to their mechanical structures [19]. The propulsion energy consumption generated by UAV flight is far greater than the communication energy consumption and computing energy consumption, so only the propulsion energy consumption of UAV flight is considered, and other energy consumption is ignored. The rate of $U_{m}$ in the *n*th time slot is expressed as follows:

(13) $$\begin{array}{c} V_{U_{m}}\left[n\right]=\left(q_{U_{m}}\left[n+1\right]-q_{U_{m}}\left[n\right]\right)/\delta ;\forall m,n=1,...,N-1 \end{array}$$

The model adopts a rotor UAV, and the total propulsion energy consumption of $U_{m}$ in the whole cycle time can be expressed as [20]

(14) $$\begin{array}{c} \begin{array}{c} E_{U_{m}}=\underset{n=1}{\sum^{N-1}}\;P_{0}\left(1+\frac{3\|V_{U_{m}}\left[n\right]{\|}^{2}}{U_{\mathrm{tip}}^{2}}\right)+\frac{1}{2}d_{r}\rho s\|V_{U_{m}}\left[n\right]{\|}^{3}+P_{i}{\left(\sqrt{1+\frac{\|V_{U_{m}}\left[n\right]{\|}^{4}}{4v_{0}^{4}}}-\frac{\|V_{U_{m}}\left[n\right]{\|}^{2}}{2v_{0}^{2}}\right)}^{1/2} \end{array} \end{array}$$

where $P_{0}$ and $P_{i}$ are the drag power and induced power in the hovering state, respectively; $U_{\mathrm{tip}}$ is the blade tip angular velocity of the UAV; $v_{0}$ is the average rotor induced velocity of the UAV in the hovering state; and $d_{r}$ and *s* are the fuselage resistance ratio and rotor firmness, respectively.

### 2.5. Problem Description

The secrecy energy efficiency is defined as the ratio of the sum of the minimum secret rate of each node of the system to the total energy consumption of the UAV. To maximize the secrecy energy efficiency (SEE) of the system, the time allocation variables, UAV trajectory, uplink transmission power, and local computing data volume are jointly optimized. This study also considers the mobility constraints, uplink transmission power constraints, time allocation constraints, computing power, and task volume constraints of two UAVs.

The optimization problem (P1) is described as follows:

(15a) $$\begin{array}{c} \left(P1\right):\max_{\tau_{I},Q_{U},P_{I},D^{\mathrm{loc}}}\;\frac{\min_{\forall l}\;B\delta \sum_{n=1}^{N}R_{l}^{\sec}\left[n\right]}{\sum_{m\in M}E_{U_{m}}} \end{array}$$

(15b) $$\begin{array}{c} \underset{l=1}{\sum^{L}}\;\tau_{I_{l}}\left[n\right]\leq 1\;\; \end{array}$$

(15c) $$\begin{array}{c} 0\leq \tau_{I_{l}}\left[n\right]\leq 1\;\; \end{array}$$

(15d) $$\begin{array}{c} \|q_{U_{m}}\left[n+1\right]-q_{U_{m}}\left[n\right]{\|}^{2}\leq V_{\max}^{2}\delta^{2} \end{array}$$

(15e) $$\begin{array}{c} q_{U_{m}}\left[1\right]=q_{U_{m}0}\;\; \end{array}$$

(15f) $$\begin{array}{c} q_{U_{m}}\left[N\right]=q_{U_{m}F}\;\; \end{array}$$

(15g) $$\begin{array}{c} 0\leq P_{I_{l}}\left[n\right]\leq P_{\max}\;\; \end{array}$$

(15h) $$\begin{array}{c} c_{I}D_{l}^{\mathrm{loc}}\left[n\right]\leq \delta F_{I}^{\max}\;\; \end{array}$$

(15i) $$\begin{array}{c} c_{U}B\delta R_{l}^{\sec}\left[n\right]\leq \delta \tau_{I_{l}}\left[n\right]F_{U}^{\max}\; \end{array}$$

(15j) $$\begin{array}{c} D_{l}^{\mathrm{loc}}\left[n\right]+B\delta R_{l}^{\sec}\left[n\right]\geq Q_{l}\; \end{array}$$

where $\left\{\tau_{I},Q_{U},P_{I},D^{\mathrm{loc}}\right\}$, respectively, represent the time allocation variables, UAV trajectory, uplink transmission power, and local calculated data volume to be optimized.

Solving problem (P1) has the following difficulties: (1) The objective function of the problem is not smooth at zero. (2) The objective function is a fractional non-convex function. (3) There are a large number of optimization variables and tight coupling between variables. (4) Multiple constraints are non-convex with respect to the optimization variables.

## 3. Joint Optimization Algorithm

A block coordinate descent (BCD) algorithm with an update rate is proposed to solve the coupling problem between variables. The successive convex approximation (SCA) (Appendix A) technique and Dinkelbach algorithm [21] are applied to solve non-convex function and fractional optimization problems. The problem that the objective function is not smooth at zero can be solved by setting $P_{m}=0$. Let $Z=\left\{\tau_{I},Q_{U},P_{I},D^{\mathrm{loc}}\right\}$ be the set of all optimization variables; then, variables except trajectory can be expressed as $Z\backslash \left\{Q_{U}\right\}$. Problem (P1) is decoupled into optimization of $Z\backslash \left\{Q_{U}\right\}$ and trajectory optimization of $Q_{U}$ and solved alternately until the algorithm converges.

### 3.1. Optimization of $Z\backslash \left\{Q_{U}\right\}$

For a given UAV trajectory $Q_{U}$, the optimization problem of $Z\backslash \left\{Q_{U}\right\}$ can be established as follows:

(16a) $$\begin{array}{c} \left(P2\right):\max_{Z\backslash \left\{Q_{U}\right\}}\;\frac{\min_{\forall l}\;B\delta \sum_{n=1}^{N}R_{l}^{\sec}\left[n\right]}{E_{\mathrm{total}}} \end{array}$$

(16b) $$\begin{array}{c} \underset{l=1}{\sum^{L}}\;\tau_{I_{l}}\left[n\right]\leq 1\;\; \end{array}$$

(16c) $$\begin{array}{c} 0\leq \tau_{I_{l}}\left[n\right]\leq 1\;\; \end{array}$$

(16d) $$\begin{array}{c} 0\leq P_{I_{l}}\left[n\right]\leq P_{\max}\;\; \end{array}$$

(16e) $$\begin{array}{c} c_{I}D_{l}^{\mathrm{loc}}\left[n\right]\leq \delta F_{I}^{\max}\;\; \end{array}$$

(16f) $$\begin{array}{c} c_{U}B\delta R_{l}^{\sec}\left[n\right]\leq \delta \tau_{I_{l}}\left[n\right]F_{U}^{\max}\; \end{array}$$

(16g) $$\begin{array}{c} D_{l}^{\mathrm{loc}}\left[n\right]+B\delta R_{l}^{\sec}\left[n\right]\geq Q_{l}\; \end{array}$$

where $E_{\mathrm{total}}=\sum_{m\in M}E_{U_{m}}$. Since the propulsion energy consumption of the UAV is independent of the node time scheduling, transmission rate, and computation task amount, $E_{\mathrm{total}}$ is a constant. We introduce slack variables {η, $s_{I}^{1}$, $s_{I}^{2}$, $\theta_{I}^{1}$, $\theta_{I}^{2}\}$ and rewrite the original problem as follows:

(17a) $$\begin{array}{c} \left(P2.1\right):\max_{Z\backslash \left\{Q_{U}\right\},\eta ,s_{I}^{1},s_{I}^{2},\theta_{I}^{1},\theta_{I}^{2}}\;\frac{\eta }{E_{\mathrm{total}}}\; \end{array}$$

(17b) $$\begin{array}{c} \left(16b\right)-\left(16e\right)\;\; \end{array}$$

(17c) $$\begin{array}{c} \eta \leq B\delta \underset{n=1}{\sum^{N}}\;s_{I_{l}}^{1}\left[n\right]-s_{I_{l}}^{2}\left[n\right]\; \end{array}$$

(17d) $$\begin{array}{c} s_{I_{l}}^{1}\left[n\right]\leq \tau_{I_{l}}\left[n\right]\theta_{I_{l}}^{1}\left[n\right]\; \end{array}$$

(17e) $$\begin{array}{c} s_{I_{l}}^{2}\left[n\right]\geq \tau_{I_{l}}\left[n\right]\theta_{I_{l}}^{2}\left[n\right]\; \end{array}$$

(17f) $$\begin{array}{c} \theta_{I_{l}}^{1}\left[n\right]\leq \log_{2}\left(1+C_{l}^{1}\left[n\right]P_{I_{l}}\left[n\right]\right) \end{array}$$

(17g) $$\begin{array}{c} \theta_{I_{l}}^{2}\left[n\right]\geq \log_{2}\left(1+C_{l,k}^{2}\left[n\right]P_{I_{l}}\left[n\right]\right) \end{array}$$

(17h) $$\begin{array}{c} c_{U}B\delta \left(s_{I_{l}}^{1}\left[n\right]-s_{I_{l}}^{2}\left[n\right]\right)\leq \delta \tau_{I_{l}}\left[n\right]F_{U}^{\max} \end{array}$$

(17i) $$\begin{array}{c} D_{l}^{loc}\left[n\right]+B\delta \left(s_{I_{l}}^{1}\left[n\right]-s_{I_{l}}^{2}\left[n\right]\right)\geq Q_{l} \end{array}$$

where $C_{l}^{1}\left[n\right]=\left(\sum_{m\in M}\;a_{m}\left[n\right]h_{I_{l}U_{m}}\left[n\right]\right)/\left(\sigma^{2}+P_{J}h_{U_{1}U_{2}}\left[n\right]\right)$ and $C_{l,k}^{2}\left[n\right]=h_{E_{k}I_{l}}/\left(\sigma^{2}+\underset{m=1}{\sum^{2}}\;\left(1-a_{m}\left[n\right]\right)P_{J}h_{U_{m}E_{k}}\left[n\right]\right)$, independent of the optimization variables of the sub-problems in this section, are constants.

Due to the introduction of slack variables, the solution obtained by problem (P2.1) is the lower bound of the optimal solution of the original problem. After *r* iterations, the time scheduling and transmission power of node $I_{l}$ in time slot *n* are defined as $\tau_{I_{l}}^{r}\left[n\right]$ and $P_{I_{l}}^{r}\left[n\right]$, respectively. Equations (17d) and (17e) can be rewritten as follows:

(18a) $$\begin{array}{c} s_{I_{l}}^{1}\left[n\right]\leq \frac{{\left(\tau_{I_{l}}\left[n\right]+\theta_{I_{l}}^{1}\left[n\right]\right)}^{2}}{4}-\frac{{\left(\tau_{I_{l}}\left[n\right]-\theta_{I_{l}}^{1}\left[n\right]\right)}^{2}}{4} \end{array}$$

(18b) $$\begin{array}{c} s_{I_{l}}^{2}\left[n\right]\geq \frac{{\left(\tau_{I_{l}}\left[n\right]+\theta_{I_{l}}^{2}\left[n\right]\right)}^{2}}{4}-\frac{{\left(\tau_{I_{l}}\left[n\right]-\theta_{I_{l}}^{2}\left[n\right]\right)}^{2}}{4} \end{array}$$

To convert the constraints in (18) into convex constraints, the second order cone (SOC) method [22] is applied, and two convex constraints in (19) are obtained, which are expressed as follows:

(19a) $$\begin{array}{c} s_{I_{l}}^{1}\left[n\right]\leq \frac{2\left(\tau_{I_{l}}^{r}\left[n\right]+\theta_{I_{l}}^{1\left(r\right)}\left[n\right]\right)\left(\tau_{I_{l}}\left[n\right]+\theta_{I_{l}}^{1}\left[n\right]\right)-{\left(\tau_{I_{l}}^{r}\left[n\right]-\theta_{I_{l}}^{1\left(r\right)}\left[n\right]\right)}^{2}}{4}-\frac{{\left(\tau_{I_{l}}\left[n\right]-\theta_{I_{l}}^{1}\left[n\right]\right)}^{2}}{4} \end{array}$$

(19b) $$\begin{array}{c} s_{I_{l}}^{2}\left[n\right]\geq \frac{{\left(\tau_{I_{l}}\left[n\right]+\theta_{I_{l}}^{2}\left[n\right]\right)}^{2}}{4}-\frac{2\left(\tau_{I_{l}}^{r}\left[n\right]-\theta_{I_{l}}^{2\left(r\right)}\left[n\right]\right)\left(\tau_{I_{l}}\left[n\right]-\theta_{I_{l}}^{2}\left[n\right]\right)}{4}+\frac{{\left(\tau_{I_{l}}^{r}\left[n\right]-\theta_{I_{l}}^{2\left(r\right)}\left[n\right]\right)}^{2}}{4} \end{array}$$

where $\theta_{I_{l}}^{1\left(r\right)}\left[n\right]=\log_{2}\left(1+C_{l}^{1}\left[n\right]P_{I_{l}}^{r}\left[n\right]\right)$, $\theta_{I_{l}}^{2\left(r\right)}\left[n\right]=\log_{2}\left(1+C_{l}^{2}\left[n\right]P_{I_{l}}^{r}\left[n\right]\right)$.

Applying the continuous convex optimization approximation algorithm to the non-convex constraints in (17g), the following expression can be obtained:

(20) $$\begin{array}{c} \theta_{I_{l}}^{2}\left[n\right]\geq M_{l,k}^{r}\left[n\right]\left(P_{I_{l}}\left[n\right]-P_{I_{l}}^{r}\left[n\right]\right)+T_{l,k}^{r}\left[n\right]\log_{2}\left(1+C_{l,k}^{2}\left[n\right]P_{I_{l}}\left[n\right]\right) \end{array}$$

where $M_{l,k}^{r}\left[n\right]=C_{l,k}^{2}\left[n\right]/\left(\log\left(2\right)\left(1+C_{l,k}^{2}\left[n\right]P_{I_{l}}^{r}\left[n\right]\right)\right)$, $T_{l,k}^{r}\left[n\right]=\log_{2}\left(1+C_{l,k}^{2}\left[n\right]P_{I_{l}}^{r}\left[n\right]\right)$.

With the above mathematical approximation, problem (P2.2) can be rewritten as follows:

$$\left(P2.2\right):\max_{Z\backslash \left\{Q_{U}\right\},\eta ,s_{I}^{1},s_{I}^{2},\theta_{I}^{1},\theta_{I}^{2}}\;\frac{\eta }{E_{\mathrm{total}}}$$

$$\left(17b\right),\;\left(17c\right),\;\left(17f\right),\;\left(17h\right),\;\left(17i\right),\;\left(19\right),\;\left(20\right)$$

Thus far, (P2.2) can be solved with the CVX tools.

### 3.2. UAV Trajectory Optimization

Given time schedule $\tau_{I}$ of sensor nodes, transmission power $P_{I}$, and local computation task $D^{\mathrm{loc}}$, the trajectory optimization sub-problem of the UAV can be expressed as follows:

(21a) $$\begin{array}{c} \left(P3\right)\max_{Q_{U}}\;\frac{\min_{\forall l}\;B\delta \sum_{n=1}^{N}R_{l}^{\sec}\left[n\right]}{\sum_{m\in M}E_{U_{m}}}\; \end{array}$$

(21b) $$\begin{array}{c} \|q_{U_{m}}\left[n+1\right]-q_{U_{m}}\left[n\right]{\|}^{2}\leq V_{\max}^{2}\delta^{2} \end{array}$$

(21c) $$\begin{array}{c} q_{U_{m}}\left[1\right]=q_{U_{m}0}\;\; \end{array}$$

(21d) $$\begin{array}{c} q_{U_{m}}\left[N\right]=q_{U_{m}F}\;\; \end{array}$$

(21e) $$\begin{array}{c} c_{U}B\delta R_{l}^{\sec}\left[n\right]\leq \delta \tau_{I_{l}}\left[n\right]F_{U}^{\max}\; \end{array}$$

(21f) $$\begin{array}{c} D_{l}^{\mathrm{loc}}\left[n\right]+B\delta R_{l}^{\sec}\left[n\right]\geq Q_{l}\; \end{array}$$

By introducing the auxiliary variables $\eta ,z_{I}^{1},z_{I}^{2},X_{I},Y_{I},\mathrm{and}\;\varphi_{I}$, the original problem can be rewritten as follows:

(22a) $$\begin{array}{c} \left(P3.1\right)\max_{Q_{U},\eta ,z_{I}^{1},z_{I}^{2},X_{I},Y,\varphi_{U}}\;\frac{\eta }{\sum_{m\in M}\sum_{n\in N}E_{U_{m}}^{1}\left[n\right]+P_{i}\varphi_{U_{m}}\left[n\right]}\; \end{array}$$

(22b) $$\begin{array}{c} \left(21b\right)-\left(21d\right)\;\;\;\; \end{array}$$

(22c) $$\begin{array}{c} \eta \leq B\delta \underset{n=1}{\sum^{N}}\;z_{I_{l}}^{1}\left[n\right]-z_{I_{l}}^{2}\left[n\right]\;\;\; \end{array}$$

(22d) $$\begin{array}{c} z_{I_{l}}^{1}\left[n\right]\leq \tau_{I_{l}}\left[n\right]\frac{1}{ln2}\left(X_{I_{l}}\left[n\right]-Y\left[n\right]\right)\;\; \end{array}$$

(22e) $$\begin{array}{c} z_{I_{l}}^{2}\left[n\right]\geq \tau_{I_{l}}\left[n\right]\log_{2}\left(1+\frac{P_{I_{l}}\left[n\right]h_{E_{k}I_{l}}}{\sigma^{2}+\sum_{m=1}^{2}\frac{\left(1-a_{m}\left[n\right]\right)P_{J}\rho_{0}}{∥q_{U_{m}}\left[n\right]-w_{E_{k}}\left[n\right]{∥}^{2}+H^{2}}}\right) \end{array}$$

(22f) $$\begin{array}{c} P_{I_{l}}\left[n\right]\left(\sum_{m\in M}\;a_{m}\left[n\right]h_{I_{l}U_{m}}\left[n\right]\right)+\sigma^{2}+P_{J}h_{U_{1}U_{2}}\left[n\right]\geq e^{X_{I_{l}}\left[n\right]} \end{array}$$

(22g) $$\begin{array}{c} P_{J}h_{U_{1}U_{2}}\left[n\right]+\sigma^{2}\leq e^{Y\left[n\right]}\;\;\; \end{array}$$

(22h) $$\begin{array}{c} \varphi_{U_{m}}^{-2}\left[n\right]\leq \varphi_{U_{m}}^{2}\left[n\right]+∥q_{U_{m}}\left[n+1\right]-q_{U_{m}}\left[n\right]{∥}^{2}/v_{0}^{2};\;\;\;\;\;\;\;\;\;\;\;\;\;\forall m,n=1,N-1 \end{array}$$

(22i) $$\begin{array}{c} c_{U}B\delta \left(z_{I_{l}}^{1}\left[n\right]-z_{I_{l}}^{2}\left[n\right]\right)\leq \delta \tau_{I_{l}}\left[n\right]F_{U}^{\max}\;\; \end{array}$$

(22j) $$\begin{array}{c} D_{l}^{\mathrm{loc}}\left[n\right]+B\delta \left(z_{I_{l}}^{1}\left[n\right]-z_{I_{l}}^{2}\left[n\right]\right)\geq Q_{l}\;\; \end{array}$$

where $E_{U_{m}}^{1}\left[n\right]=P_{0}\left(1+\frac{3\|V_{U_{m}}\left[n\right]{\|}^{2}}{U_{tip}^{2}}\right)+\frac{1}{2}d_{r}\rho s\|V_{U_{m}}\left[n\right]{\|}^{3}$.

For the constraints in (22e), regarding $∥q_{U_{m}}\left[n\right]-w_{E_{k}}\left[n\right]{∥}^{2}+H^{2}$ as a whole, suppose $u_{U_{m}E_{k}}\left[n\right]=∥q_{U_{m}}\left[n\right]-w_{E_{k}}\left[n\right]{∥}^{2}+H^{2}$. The right-hand function of (22e) is a concave function about $u_{U_{m}E_{k}}\left[n\right]$, and its upper bound can be obtained by the first-order Taylor expansion at this point. Let the trajectory of the UAV in the *n*th time slot after *r* iterations be $q_{U_{m}}^{r}\left[n\right]$. Expression (22e) can be transformed into the following convex constraints:

(23) $$\begin{array}{c} z_{I_{l}}^{2}\left[n\right]\geq \tau_{I_{l}}\left[n\right]\left(\underset{m=1}{\sum^{M}}\;F_{I_{l}U_{m}E_{k}}^{r}\left[n\right]\left(u_{U_{m}E_{k}}\left[n\right]-u_{U_{m}E_{k}}^{r}\left[n\right]\right)+X_{I_{l}E_{k}}^{r}\left[n\right]\right) \end{array}$$

where $F_{I_{l}U_{m}E_{k}}^{r}\left[n\right]=\frac{\rho_{0}\left(1-a_{m}\left[n\right]\right)P_{J}P_{I_{l}}\left[n\right]h_{E_{k}I_{l}}}{u_{U_{m}E_{k}}^{2\left(r\right)}\left[n\right]\left(\sum_{M}\frac{\rho_{0}P_{J}\left(1-a_{m}\left[n\right]\right)}{u_{U_{m}E_{k}}^{r}\left[n\right]\sigma^{2}}+\sigma^{2}+P_{I_{l}}\left[n\right]h_{E_{k}I_{l}}\right)\left(\sum_{M}\frac{\rho_{0}P_{J}\left(1-a_{m}\left[n\right]\right)}{u_{U_{m}E_{k}}^{r}\left[n\right]\sigma^{2}}+\sigma^{2}\right)}$ and $X_{I_{l}E_{k}}^{r}\left[n\right]=\tau_{I_{l}}\left[n\right]\log_{2}\left(1+\frac{P_{I_{l}}\left[n\right]h_{E_{k}I_{l}}}{\sigma^{2}+\sum_{m=1}^{2}\frac{\left(1-a_{m}\left[n\right]\right)P_{J}\rho_{0}/\sigma^{2}}{∥q_{U_{m}}^{r}\left[n\right]-w_{E_{k}}\left[n\right]{∥}^{2}+H^{2}}}\right)$.

Likewise, applying continuous convex optimization approximations to $h_{I_{l}U_{m}}\left[n\right]$ and $h_{U_{1}U_{2}}\left[n\right]$ in (22f), their non-convex lower bounds are obtained as follows:

(24a) $$\begin{array}{c} h_{I_{l}U_{m}}^{lb}\left[n\right]=\frac{2\rho_{0}/\sigma^{2}}{∥q_{U_{m}}^{r}\left[n\right]-w_{I_{l}}{∥}^{2}+H^{2}}-\frac{\rho_{0}\left(∥q_{U_{m}}\left[n\right]-w_{I_{l}}{∥}^{2}+H^{2}\right)}{\sigma^{2}{\left(∥q_{U_{m}}^{r}\left[n\right]-w_{I_{l}}{∥}^{2}+H^{2}\right)}^{2}} \end{array}$$

(24b) $$\begin{array}{c} h_{U_{1}U_{2}}^{lb}\left[n\right]=\frac{2\rho_{0}/\sigma^{2}}{∥q_{U_{1}}^{r}\left[n\right]-q_{U_{2}}^{r}{∥}^{2}}-\frac{\rho_{0}\left(∥q_{U_{1}}\left[n\right]-q_{U_{2}}\left[n\right]{∥}^{2}\right)}{\sigma^{2}{\left(∥q_{U_{1}}^{r}\left[n\right]-q_{U_{2}}^{r}\left[n\right]{∥}^{2}\right)}^{2}}\; \end{array}$$

Then, (22f) can be replaced by (25).

(25) $$\begin{array}{c} P_{I_{l}}\left[n\right]\left(\sum_{m\in M}\;a_{m}\left[n\right]h_{I_{l}U_{m}}^{lb}\left[n\right]\right)+\sigma^{2}+P_{J}h_{U_{1}U_{2}}^{lb}\left[n\right]\geq e^{X_{I_{l}}\left[n\right]} \end{array}$$

To solve the non-convex problem in (22g), introduce auxiliary variables $\omega_{U_{1}U_{2}}\left[n\right]$ and replace it with (26).

(26a) $$\begin{array}{c} P_{J}\frac{\rho_{0}}{\sigma^{2}\omega_{U_{1}U_{2}}\left[n\right]}+\sigma^{2}\leq e^{Y\left[n\right]}\; \end{array}$$

(26b) $$\begin{array}{c} \omega_{U_{1}U_{2}}\left[n\right]\leq ∥q_{U_{1}}\left[n\right]-q_{U_{2}}\left[n\right]{∥}^{2} \end{array}$$

The following convex constraints can be obtained by applying first-order Taylor expansion to the right side of the two equations:

(27a) $$\begin{array}{c} P_{J}\frac{\rho_{0}}{\sigma^{2}\omega_{U_{1}U_{2}}\left[n\right]}+\sigma^{2}\leq e^{Y^{r}\left[n\right]}\left(Y\left[n\right]-Y^{r}\left[n\right]+1\right)\;\; \end{array}$$

(27b) $$\begin{array}{c} \omega_{U_{1}U_{2}}\left[n\right]\leq 2{\left(q_{U_{1}}^{r}\left[n\right]-q_{U_{2}}^{r}\left[n\right]\right)}^{T}\left(q_{U_{1}}\left[n\right]-q_{U_{2}}\left[n\right]\right)-∥q_{U_{1}}^{r}\left[n\right]-q_{U_{2}}^{r}\left[n\right]{∥}^{2} \end{array}$$

Similarly, (22h) can be approximated as

(28) $$\begin{array}{c} \varphi_{U_{m}}^{-2}\left[n\right]\leq \varphi_{U_{m}}^{2\left(r\right)}\left[n\right]+2\varphi_{U_{m}}^{r}\left[n\right]\left(\varphi_{U_{m}}\left[n\right]-\varphi_{U_{m}}^{r}\left[n\right]\right)-\;\;\;\;\;\;\\ \frac{∥q_{U_{m}}^{r}\left[n+1\right]-q_{U_{m}}^{r}\left[n\right]{∥}^{2}+2{\left(q_{U_{m}}^{r}\left[n+1\right]-q_{U_{m}}^{r}\left[n\right]\right)}^{T}\left(q_{U_{m}}\left[n+1\right]-q_{U_{m}}\left[n\right]\right)}{v_{0}^{2}};\\ \forall m,n=1,\ldots ,N-1\;\;\;\;\; \end{array}$$

After the above approximation, problem (P3.1) can be rewritten as follows:

(29a) $$\begin{array}{c} \left(P3.2\right)\max_{Q_{U},\eta ,z_{I}^{1},z_{I}^{2},X_{I},Y,\varphi_{U},\omega }\;\frac{\eta }{\sum_{m\in M}\sum_{n\in N}E_{U_{m}}^{1}\left[n\right]+P_{i}\varphi_{U_{m}}\left[n\right]} \end{array}$$

(29b) $$\begin{array}{c} \left(22b\right)-\left(22d\right),\left(22i\right),\left(22j\right),\left(23\right),\left(25\right),\left(27\right),\left(28\right)\; \end{array}$$

Problem (P3.2) can be further solved by Dinkelbach’s algorithm. First, $\mu^{*}\;$ is defined as the maximum value of the system SEE, and $E_{\mathrm{total}}=\sum_{m\in M}\sum_{n\in N}\;E_{U_{m}}^{1}\left[n\right]+P_{i}\varphi_{U_{m}}\left[n\right]$. Problem (P3.2) can be equivalent to iteratively solving the following problem:

(30a) $$\begin{array}{c} \left(P3.3\right)\max_{Q_{U},\eta ,z_{I}^{1},z_{I}^{2},X_{I},Y,\varphi_{U},\omega }\;\eta -\mu^{*}E_{\mathrm{total}} \end{array}$$

(30b) $$\begin{array}{c} \left(29b\right)\;\;\; \end{array}$$

Problem (P3.3) can be solved with the CVX tools. The logic of the algorithm is shown in Algorithm 1. Due to the introduction of slack variables, the result obtained from the solution is the lower bound of the original problem.
**Algorithm 1** Trajectory Planning Algorithm for Problem (P3).1:Initialization: Initial trajectory of the base station UAV is $Q_{U}^{0}$, initial value of energy efficiency μ=0, maximum threshold ϵ>0, and number of iterations r=0.2:Solve problem (P2.3) given UAV trajectory $Q_{U}^{r}$ and system secret energy efficiency value μ, and obtain optimal solutions $Q_{U}^{*}$, $\eta^{*}$, and $z_{I}^{1\left(*\right)},z_{I}^{2\left(*\right)},X_{I}^{*},Y_{I}^{*},\varphi_{U}^{*},\;\mathrm{and}\;{\omega^{*}}_{I}$.3:Calculate $\eta^{*}-\mu E_{\mathrm{total}}^{*}$. If $\eta^{*}-\mu E_{\mathrm{total}}^{*}<\epsilon$, then the algorithm converges, obtains the optimal solution, and outputs $Q_{U}^{*}$, $\eta^{*}$, $z_{I}^{1\left(*\right)},z_{I}^{2\left(*\right)},X_{I}^{*},Y_{I}^{*},\varphi_{U}^{*},{\omega^{*}}_{I}$. Otherwise, continue with steps 4–5.4:Make $\mu^{*}=\frac{\eta^{*}}{E_{\mathrm{total}}^{*}}$, update parameters $\mu =\mu^{*}$, and update UAV trajectory $Q_{U}^{r+1}=Q_{U}^{*}$.5:Update the number of iterations r=r+1. Then, execute step 3 again to check whether the algorithm converges.

### 3.3. Overall Algorithm Description

To solve problem (P1), the updated-rate assisted block coordinate descent algorithm is applied. The sub-problems in the previous two sections are solved alternately, and the optimal solution of the original problem is obtained. The overall idea is as follows: decouple the original problem into two sub-problems: variable $Z\backslash \left\{Q_{U}\right\}$ optimization and trajectory optimization. Then, fix one set of variables to solve sub-problems (P2.2) and (P3.3) alternately. The optimal solution is used as the reference value for the next iteration.

The overall description of the joint optimization algorithm is shown in Algorithm 2.
**Algorithm 2** Joint Optimization Algorithm for Maximizing Energy Efficiency1:Initialize variables $\tau_{I}^{0}$, $Q_{U}^{0}$, $P_{I}^{0}$, and $D^{\mathrm{loc}\left(0\right)}$. Set the minimum error value ϵ>0. Let the number of iterations r=0, update rate ξ=1, and update factor ε=0.1.2:Given $Q_{U}^{r}$ conditions, use the continuous convex optimization approximation algorithm to solve problem (P2), obtain optimal solutions $\tau_{I}^{*}$, $P_{I}^{*}$, $D^{\mathrm{loc}\left(*\right)}$, and let $\tau_{I}^{r+1}=\xi \left(\tau_{I}^{*}-\tau_{I}^{r}\right)+\tau_{I}^{r}$, $P_{I}^{r+1}=\xi \left(P_{I}^{*}-P_{I}^{r}\right)+P_{I}^{r}$, $D^{\mathrm{loc}\left(r+1\right)}=\xi \left(D_{I}^{*}-D_{I}^{r}\right)+D_{I}^{r}$.3:Given $\tau_{I}^{r+1}$, $P_{I}^{r+1}$, and $D^{\mathrm{loc}\left(r+1\right)}$, Algorithm 1 is used to solve problem (P3), obtain the optimized relay UAV trajectory $Q_{U}^{*}$, and let $Q_{U}^{r+1}=\xi \left(Q_{I}^{*}-Q_{I}^{r}\right)+Q_{I}^{r}$.4:Update the number of iterations r=r+1, $\xi =\xi /\left(1+\left(r-1\right)\times \varepsilon \right)$.5:Calculate the increment of the target value Δ. If Δ<ϵ, then the algorithm converges and ends. Otherwise, continue with steps 2–4.6:Output the optimized solution $\tau_{I}^{*}$, $Q_{U}^{*}$, $P_{I}^{*}$, $D^{\mathrm{loc}\left(*\right)}$, and the maximum secret energy efficiency.

In Algorithm 2, each sub-problem can be approximated as a linear problem by first-order Taylor expansion. Then the interior point method is used to solve problems (P2), and the computational complexity is $O\left(\sqrt{\tilde{N}}\log\frac{1}{\varepsilon_{0}}\right)$, where $\tilde{N}$ represents the number of decision variables [16], and $\varepsilon_{0}$ indicates iteration accuracy. Similarly, the computational complexity of questions (P3) is $O\left(C\sqrt{\tilde{N}}\log\frac{1}{\varepsilon_{0}}\right)$, where *C* represents the number of iterations for the update of μ in Algorithm 1. Therefore, the overall computational complexity of Algorithm 2 is $O\left(\sqrt{\left(5L+1\right)N}\log\frac{1}{\varepsilon_{0}}\right)+O\left(C\sqrt{\left(2L+1\right)N+1}\log\frac{1}{\varepsilon_{0}}\right)$ [23].

Statements (P2.2) and (P3.3) are the approximate problem of the original sub-problems. Therefore, we prove that Algorithm 2 can converge as follows. Define

(31) $$\begin{array}{c} \begin{array}{c} \chi_{\tau_{I}}^{r\left(lb\right)}\left(\tau_{I},Q_{U},P_{I},D^{\mathrm{loc}}\right)=\chi_{\tau_{I}}^{r}\\ \;\chi_{Q_{U}}^{r\left(lb\right)}\left(\tau_{I},Q_{U},P_{I},D^{\mathrm{loc}}\right)=\chi_{Q_{U}}^{r}\\ \begin{array}{c} \chi_{P_{I}}^{r\left(lb\right)}\left(\tau_{I},Q_{U},P_{I},D^{\mathrm{loc}}\right)=\chi_{P_{I}}^{r}\\ \;\;\;\chi_{D^{\mathrm{loc}}}^{r\left(lb\right)}\left(\tau_{I},Q_{U},P_{I},D^{\mathrm{loc}}\right)=\chi_{D^{\mathrm{loc}}}^{r} \end{array} \end{array} \end{array}$$

where $\chi_{\tau_{I}}^{r},\chi_{Q_{U}}^{r},\chi_{P_{I}}^{r},\chi_{D^{\mathrm{loc}}}^{r}$ are values of the optimal solution in Problems (P2.2) and (P3.3), respectively based on $\tau_{I},Q_{U},P_{I},D^{\mathrm{loc}}$. In Step 2 of Algorithm 2, it follows that:

(32) $$\begin{array}{c} \begin{array}{c} \chi \left(\tau_{I},Q_{U},P_{I},D^{\mathrm{loc}}\right)\overset{\left(a\right)}{=}\;\chi_{\tau_{I}}^{r\left(lb\right)}\left(\tau_{I}^{r},Q_{U}^{r},P_{I}^{r},D^{locr}\right)\\ \;\;\;\;\;\;\;\;\;\;\;\;\;\;\;\;\;\;\;\;\;\;\;\;\;\;\;\;\;\;\;\;\;\;\;\;\;\overset{\left(b\right)}{\leq }\;\chi_{\tau_{I}}^{r\left(lb\right)}\left(\tau_{I}^{r+1},Q_{U}^{r},P_{I}^{r},D^{locr}\right)\\ \;\;\;\;\;\;\;\;\;\;\;\;\;\;\;\;\;\;\;\;\;\;\;\;\;\;\;\;\;\;\overset{\left(c\right)}{\leq }\;\chi \left(\tau_{I}^{r+1},Q_{U}^{r},P_{I}^{r},D^{locr}\right) \end{array} \end{array}$$

where (a) holds because the first-order Taylor expansions in (19) are tight at the given local points, respectively; (b) holds since that given $\tau_{I}^{r+1},Q_{U}^{r},P_{I}^{r},D^{locr}$, the optimal solution $\tau_{I}^{r+1}$ is obtained by solving Problem (P2.2); (c) holds because the optimal solution of (P2.2) is lower bounded by (P2) at $\tau_{I}^{r+1}$. According to the inequality above, although only one approximate optimization problem (P2.2) is solved, the target value of Problem (P2) is still non decreasing after each iteration. Second, in step 2 of Algorithm 2, given $\tau_{I}^{r+1},Q_{U}^{r},P_{I}^{r},D^{locr}$, it follows that:

(33) $$\begin{array}{c} \chi \left(\tau_{I}^{r+1},Q_{U}^{r},P_{I}^{r},D^{locr}\right)\overset{\left(d\right)}{\leq }\;\chi \left(\tau_{I}^{r+1},Q_{U}^{r},P_{I}^{r+1},D^{locr}\right) \end{array}$$

where (d) holds, since that by given $\tau_{I}^{r+1},Q_{U}^{r},P_{I}^{r},D^{locr}$, (P2) can be handled with optimal solution $P_{I}^{r+1}$. Then, in step 2 of Algorithm 2, for the given $\tau_{I}^{r+1},Q_{U}^{r},P_{I}^{r+1},D^{locr}$, it follows that:

(34) $$\begin{array}{c} \chi \left(\tau_{I}^{r+1},Q_{U}^{r},P_{I}^{r+1},D^{locr}\right)\overset{\left(e\right)}{\leq }\;\chi \left(\tau_{I}^{r+1},Q_{U}^{r},P_{I}^{r+1},D^{locr+1}\right) \end{array}$$

Then, in step 3 of Algorithm 2, for the given $\tau_{I}^{r+1},Q_{U}^{r},P_{I}^{r+1},D^{locr+1}$, it follows that:

(35) $$\begin{array}{c} \chi \left(\tau_{I}^{r+1},Q_{U}^{r},P_{I}^{r+1},D^{locr+1}\right)\overset{\left(f\right)}{\leq }\;\chi \left(\tau_{I}^{r+1},Q_{U}^{r+1},P_{I}^{r+1},D^{locr+1}\right) \end{array}$$

Based on the above inequality, we obtain:

(36) $$\begin{array}{c} \chi \left(\tau_{I}^{r},Q_{U}^{r},P_{I}^{r},D^{locr}\right)\leq \chi \left(\tau_{I}^{r+1},Q_{U}^{r+1},P_{I}^{r+1},D^{locr+1}\right) \end{array}$$

It indicates that the target value of Problem (P1) is non decreasing after each iteration of Algorithm 2. Since the objective value of the problem (P1) is bounded by a finite value, Algorithm 2 must converge to a stable point.

## 4. Analysis of Simulation Results

In this work, a large number of simulation experiments were carried out to verify the effectiveness of the proposed joint optimization scheme based on multifunctional UAVs in improving the security energy efficiency of the system. First, the parameter settings of the simulation experiment are explained, and then part of the optimization results are shown. Finally, the convergence performance, system security energy efficiency performance, and security rate performance of the proposed joint optimization algorithm are given, and the effectiveness of the proposed joint optimization algorithm is verified.

### 4.1. Simulation Parameter Settings

The settings of each parameter in the simulation experiment are listed in Table 2.

### 4.2. Performance Simulation Results

**Figure 2:**
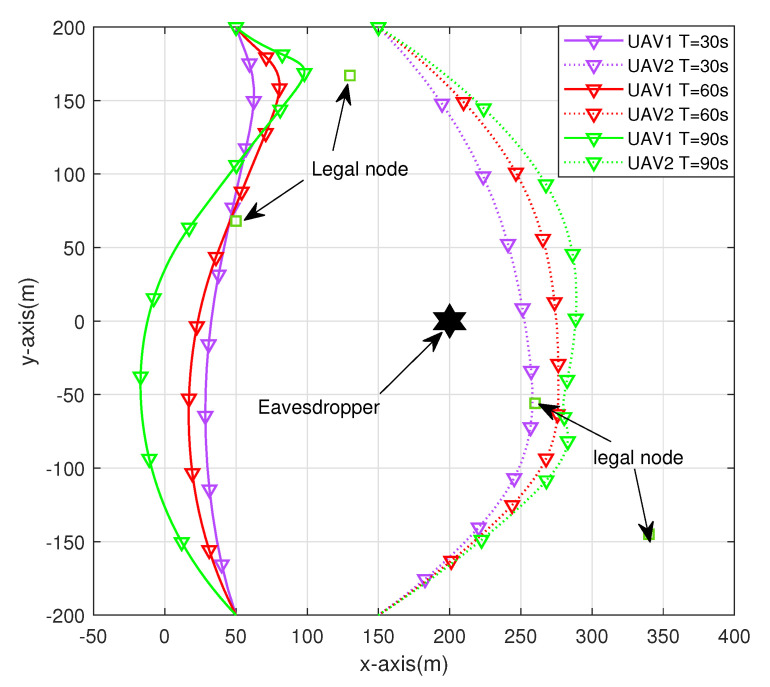
UAV trajectories at different cycle times.

Figure 2 shows the trajectories of two UAVs obtained through the joint optimization algorithm at different cycle times. The positions of four ground sensor nodes and eavesdropping nodes are marked in the figure. We choose to observe the UAV track under three cycle times of T = 30 s, T = 60 s, and T = 90 s. These three situations can represent three actual scenarios: insufficient UAV task time, appropriate UAV task time, and sufficient UAV task time. Comparing the three different cycle times, it can be found that with the increase in the cycle time, the tendency of the auxiliary computing UAV to approach the sensor node and actively interfere with the UAV away from the sensor node is more obvious. To ensure system security energy efficiency, UAVs have more time to obtain a better channel, thus improving the system security transmission rate.

**Figure 3:**
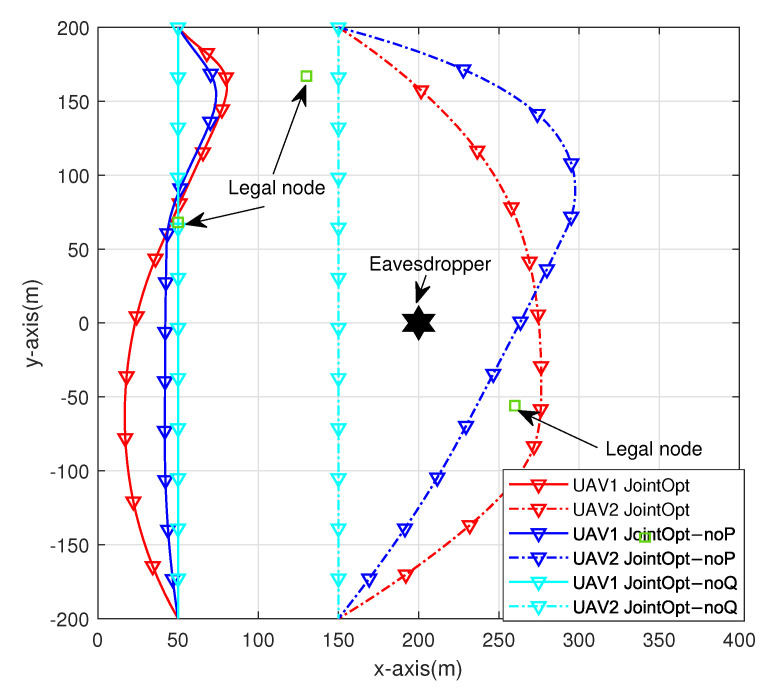
UAV trajectories of different schemes.

Figure 3 shows the trajectory optimization results of the two UAVs under the joint optimization scheme and two other benchmark schemes: the optimization scheme without sensor node transmission power optimization (hereinafter referred to as the noP optimization scheme) and the optimization scheme without UAV trajectory optimization (hereinafter referred to as the noQ optimization scheme) when T = 60. A comparison of the three schemes shows that the noQ optimization scheme greatly limits the maneuverability of the UAV and causes huge losses to the performance of the system. The noP scheme also restricts the trajectory of the UAV to a certain extent. In summary, the joint optimization algorithm is of great help to improve the security and energy efficiency of the system.

**Figure 4:**
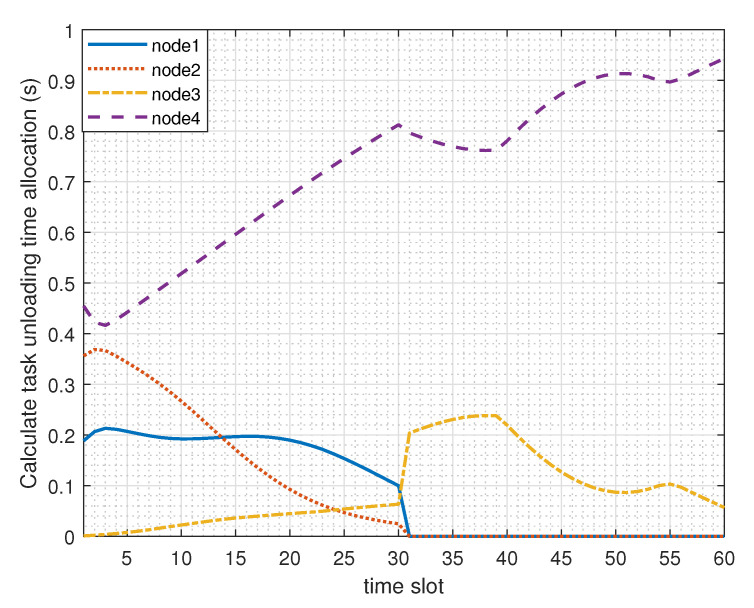
Time scheduling curve of each user in each time slot.

Figure 4 shows the time allocation and scheduling of the four nodes in each time slot when the UAV mission time is T = 60 s. Nodes 1–4 in the figure represent four ground nodes. The four nodes are divided into two situations. The first is a situation where the link confidentiality performance represented by Nodes 1, 2, and 3 needs to be maintained, and the second is a situation where the eavesdropper threat represented by Node 4 is low. It can be seen that the time scheduling coefficient of each time slot of Node 4 across the whole cycle time is within the range of [0.4,1]. As the information leakage risk of the node is low, part of the computation task can be offloaded to the auxiliary computation UAV even if the transmission channel condition is general. The three nodes in the first case are closely related to the trajectory and working mode of the UAV. Each index of the joint optimization algorithm affects the other, and the time scheduling coefficient is closely related to the channel condition of legitimate links and eavesdropping links.

### 4.3. Performance Comparison of Different Optimization Algorithms

**Figure 5:**
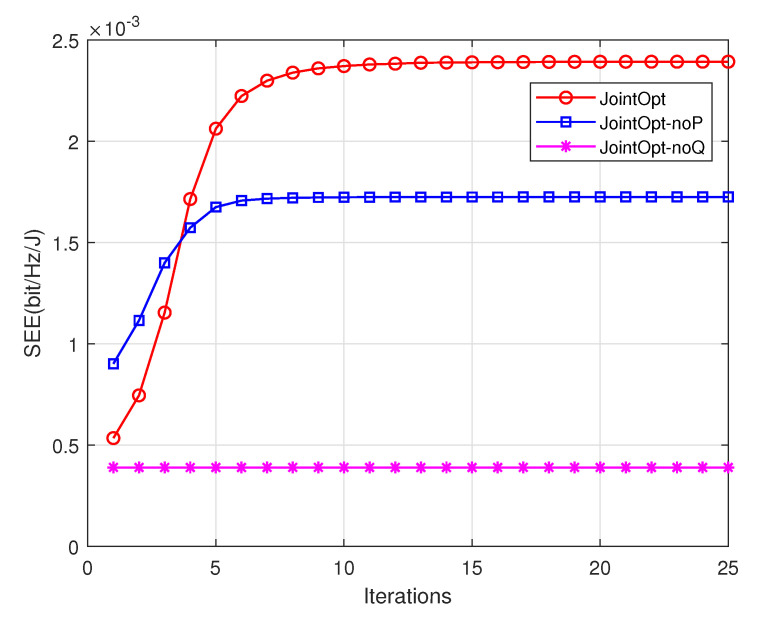
Convergence diagram of joint optimization algorithm.

Figure 5 shows the convergence of the proposed joint optimization Algorithm 2 and benchmark algorithm when the cycle time T=60 s. Compared with the benchmark scheme, the SEE of the scheme is improved by 38.5%. Figure 5 shows that the SEE of the system increases with the number of iterations, and the algorithm converges after approximately 14 iterations.

**Figure 6:**
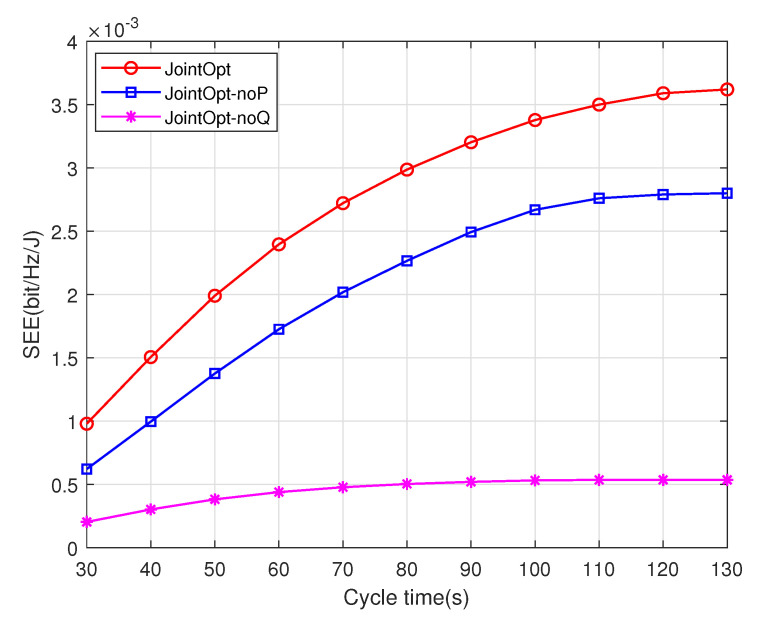
Comparison of system SEE performance.

**Figure 7:**
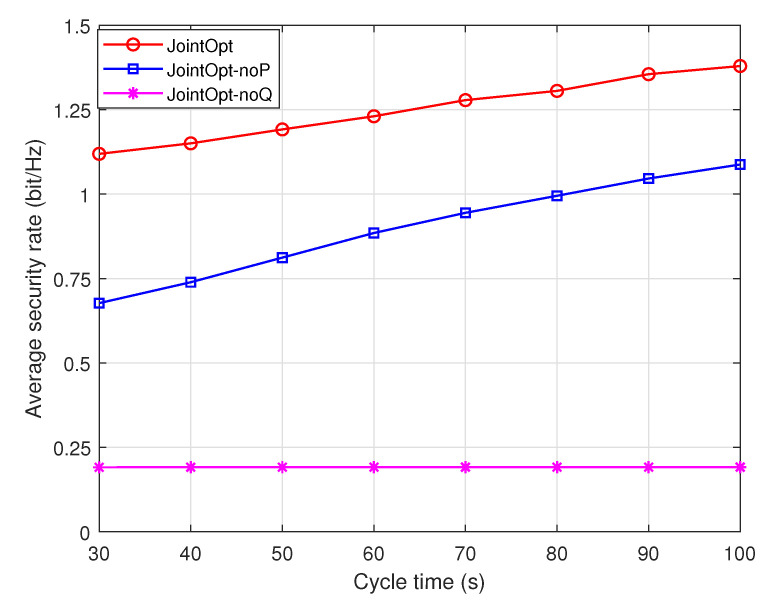
System security rate performance comparison.

Figure 6 and Figure 7 show the curves of the system security energy efficiency and security transmission rate performance versus the UAV task time T under different schemes. The simulation results show that compared with the noP optimization scheme and the noQ optimization scheme of the benchmark schemes, the proposed joint optimization scheme can achieve better performance of the system, and it is an effective means to improve the security performance of the system while taking into account the energy efficiency. Under the two performance metrics of system security energy efficiency and security transmission rate, the performance ranking of the three schemes is joint optimization scheme>noP optimization scheme>noQ optimization scheme. In addition, it can be found from the curve trends in the two figures that, with the increase in cycle time, the security transmission rate performance of the system increases linearly, whereas the security energy efficiency of the system increases slowly. Therefore, it can be concluded that blindly increasing the task time will not only improve the system transmission rate, but also bring more energy consumption, and the system energy efficiency will gradually become stable.

## 5. Conclusions

The security energy efficiency of a mobile edge computing system based on a multifunctional UAV was studied. According to the channel environment of the location, the UAV selects a working mode between auxiliary calculation and active interference to maximize the offloading rate of legal links on the premise of ensuring channel security. Under the conditions of time scheduling constraints, UAV maneuverability constraints, uplink maximum transmit power, and computational performance constraints, a block coordinate descent algorithm based on the update rate, continuous convex optimization approximation algorithm, and Dinkelbach algorithm were used to jointly optimize the time scheduling, transmission rate, task amount, and UAV trajectory of each node in order to maximize the security energy efficiency of the system. The simulation results verified the rationality and effectiveness of the joint optimization scheme.

## Figures and Tables

**Table 1:** Summary of symbols.

Symbol	Description
*L*	number of ground legal sensors
*K*	number of ground eavesdropping nodes
*H*	altitude of UAVs
$q_{U_{m}},m=\left\{1,2\right\}$	horizontal coordinate of UAV
$w_{I_{l}}$	coordinate of ground sensor nodes
$w_{E_{k}}$	coordinate of ground eavesdropping nodes
δ	timeslot
$\rho_{0}$	channel power gain
$P_{S}$	transmit power of source node
$P_{R_{\max}}$	maximum power of relay UAV
$P_{J_{\max}}$	maximum power of jamming UAV
$c_{I}$	CPU required for unit data of node
$c_{U}$	CPU required for unit data of UAV
$F_{I}^{\max}$	maximum CPU of node
$F_{U}^{\max}$	maximum CPU of UAV
*B*	channel bandwidth
$Q_{I}$	node tasks
$U_{\mathrm{tip}}$	blade tip angular velocity of the UAV
*s*	rotor firmness
ρ	air density
*A*	rotor disc area
*R*	rotor radius
*k*	inductive power increment correction factor
σ	section resistance coefficient
*W*	aircraft weight
Ω	blade angular velocity

**Table 2:** Simulation parameters.

Symbol	Value	Symbol	Value
*M*	2	$c_{I}$	1000
*L*	4	$c_{U}$	1000
*K*	1	$F_{I}^{\max}$	$10^{9}$
*B*	$10^{6}$	$F_{U}^{\max}$	$10^{10}$
*H*	50m	$Q_{I}$	$5\times 10^{5}$
$q_{U_{m}0},m=\left\{1,2\right\}$	${\left(50,200\right)}^{T}$,${\left(150,200\right)}^{T}$	$U_{\mathrm{tip}}$	60 m/s
$q_{U_{m}F},m=\left\{1,2\right\}$	${\left(50,-200\right)}^{T}$,${\left(150,-200\right)}^{T}$	$v_{0}$	2.4868 m/s
$w_{I_{l}}$	${\left(50,68\right)}^{T}$,${\left(130,167\right)}^{T}$,${\left(260,-56\right)}^{T}$,${\left(340,-145\right)}^{T}$	$d_{0}$	1
$w_{E_{k}}$	${\left(200,0\right)}^{T}$	*s*	0.0832
$V_{\max}$	40 m/s	ρ	1.225 km/m^3^
δ	1s	*A*	0.2827 m^2^
$\sigma^{2}$	−110 dBm	*R*	0.3 m
$\rho_{0}$	−60 dBm	*k*	0.1
$P_{S}$	30 dBm	σ	0.012
$P_{R_{\max}}$	20 dBm	*W*	4 N
$P_{J_{\max}}$	10 dBm	Ω	200 rad/s

## Data Availability

The data presented in this study are available in Table 2.

## Abbreviations

The following abbreviations are used in this manuscript:

UAV	Unmanned Aerial Vehicles
BCD	Block Coordinate Descent
LOS	Line-of-Sight
MEC	Mobile Edge Computing
SEE	Secrecy Energy Efficiency
SCA	Successive Convex Approximation
SOC	Second Order Cone

## Appendix A. SCA Algorithm

Through continuous convex optimization approximation, the non-convex function in the original problem can be transformed into its convex upper bound or concave lower bound, and finally the sub-optimal solution of the original problem can be obtained through multiple iterations. In each iteration of continuous convex optimization approximation, there needs to be a set of known feasible solutions, which are generally the optimal solutions obtained in the last iteration. For the first iteration, you need to set a set of initial values, and the selection of initial values will also affect the convergence speed of the iteration. The common method for boundary transformation of non-convex functions is first-order Taylor formula. If $f\left(x\right)$ is a convex function of variable *x*, then for any given initial point $x_{0}$ has

(A1) $$\begin{array}{c} f\left(x\right)\geq f\left(x_{0}\right)+\nabla f\left(x_{0}\right)\left(x-x_{0}\right) \end{array}$$

That is, the first order Taylor expansion of $f\left(x\right)$ at the initial point $x_{0}$ is its affine lower bound. Similarly, if $f\left(x\right)$ is a concave function of variable *x*, the affine upper bound of $f\left(x\right)$ can be obtained.
